# Supplementary material for: A multicenter proof-of-concept study on deep learning-based intraoperative discrimination of primary central nervous system lymphoma
Source: Nat Commun. 2024 May 4;15:3768. doi: 10.1038/s41467-024-48171-x (PMC11069536; doi:10.1038/s41467-024-48171-x)
Supplement: Supplementary file 1 — Supplementary information [file 41467_2024_48171_MOESM1_ESM.pdf]

A multicenter proof-of-concept study on deep learning-based intraoperative discrimination of primary central nervous system lymphoma

Supplementary Methods

Study participants

The internal cohort encompassed 172 H&E-stained whole-slide images (WSIs) derived from 79 patients with primary central nervous system lymphoma (PCNSL) and 597 WSIs from 353 patients diagnosed with glioma at our center, forming the basis for developing the LGNet model. External Cohort 1 comprised 300 tumor WSIs collected from 300 patients, consisting of 49 PCNSL cases and 251 glioma cases from external centers. External Cohort 2 encompassed 386 tumor WSIs sourced from 386 patients, with 364 glioma cases and 22 PCNSL cases. A proof-of-concept study involved 68 H&E-stained WSIs originating from 7 PCNSL patients and 61 glioma patients. External Cohort 1, External Cohort 2, and the proof-of-concept cohort were utilized to validate the LGNet model. Additionally, we gathered supplementary data, including 37 WSIs from 36 patients with medulloblastoma, 8 WSIs from 6 patients with central neurocytoma, 105 WSIs from 99 patients with brain metastatic cancer, and 30 WSIs from 23 patients with brain inflammation lesions at Sun Yat-sen University Cancer Center, serving as supplemental data to the internal cohort. Moreover, 41 WSIs from 41 patients with medulloblastoma, 26 WSIs from 26 patients with central neurocytoma, 51 WSIs from 51 patients with brain metastatic cancer, and 17 WSIs from 17 patients with brain inflammation lesions at Zhujiang Hospital of Southern Medical University were considered as supplemental data for External Cohort 1. Likewise, 24 WSIs from 24 patients with medulloblastoma, 12 WSIs from 12 patients with central neurocytoma, 24 WSIs from 24 patients with brain metastatic cancer, and 20 WSIs from 20 patients with brain inflammation lesions at Nanfang Hospital of Southern Medical University served as supplemental data for External Cohort 2. The internal dataset, comprising PCNSL, glioma, and other brain lesions from Sun Yat-sen University Cancer Center, facilitated model development for distinguishing PCNSL from non-PCNSL, while external test datasets from Zhujiang Hospital of Southern Medical University (External Test Dataset 1) and Nanfang Hospital of Southern Medical University and The First Affiliated Hospital of Sun Yat-sen University (External Test Dataset 2) were employed for model validation. All enrolled patients met the following criteria: (1) confirmed diagnosis of PCNSL, glioma, or other lesions through immunohistochemical and molecular testing of formalin-fixed paraffin-embedded (FFPE) samples, and (2) availability of H&E-stained frozen samples.

We excluded scanned slides exhibiting poor quality, such as those with focus issues, suboptimal staining, or evident tissue folds.

WSI preprocessing

In this study, we opted for a 10× magnification level to ensure better recognition of distinguishing features between PCNSL and glioma or non-PCNSL. Due to the extensive size of whole-slide images (WSIs), we partitioned the slides into non-overlapping 512x512 pixel windows using the openslide library. Grayscale thumbnails were generated for each slide to precisely delineate the boundaries between tissue and background regions, employing the Otsu's algorithm[1]. Tiles covering less than 30% of the surface area by tissue were identified as background and excluded, while the remaining tissue tiles were utilized in developing the deep learning model, including LGNet, and the model used in distinguishing between PCNSL and non-PCNSL. The tumor label for each tile directly corresponded to the ground-truth label of the respective WSI. Given that most frozen sections diagnosed as PCNSL and non-PCNSL contained a high proportion of tumor tissues, paracancerous tissue within the slide, potentially mixed with varying numbers of tumor cells due to the invasive border of brain tumors, was also labeled as tumors. However, tiles corresponding to paracancerous tissue were considered data with noisy labels during model training. To mitigate color variations[2] introduced by different sample preparation and staining processes at various healthcare centers, the color distribution within the tissue regions was normalized using the Vahadane method before model training. Employing diverse data augmentation techniques like color jitter, rotation, and random flipping, we enhanced the model's robustness and augmented the training data. Finally, two 224×224 pixel tiles were randomly extracted from each 512x512 tile, constituting the model's ultimate input during training, thus enriching the dataset for robust learning.

Deep learning model development

All the tiles tagged with a ground truth label that was consistent with each slide such as either PCNSL or glioma and either PCNSL or non-PCNSL were inputted into the classifiers, trained by iterative draw and rank sampling to calculate a prediction score for each tile, and their predictive labels (LGNet: 1 for 'PCNSL', 0 for 'glioma'; the model in discriminating PCNSL from non-PCNSL: 1 for 'PCNSL', 0 for 'non-PCNSL') were served as the expected outputs of the classifiers. The stochastic

gradient descent (SGD) optimizer with batch size 64 and weight decay 0.0005 was used to train each individual classifier for maximally 100 epochs. The learning rate started from 0.05 and changed with a cosine annealing schedule. It has been consistently noted that classifier training converged after approximate 100 epochs. Such training process, with a different fold as the internal validation set each time, was carried out repeatedly five times to generate correspondingly five well-trained individual classifiers, which were ensembled to form the deep learning model at the softmax layer of individual classifiers, i.e., the average probability outputs of the five individual classifiers were considered as the final prediction of the ensemble deep learning model. Since the ensemble deep learning model was responsible for classification of either PCNSL or glioma and either PCNSL or non-PCNSL based on tile-level probability rather than slide-level probability, predictive probabilities of the LGNet based on tile-level though taking their average value were aggregated into a slide-level probability, which was further averaged to calculate a patient-level probability if a patient had more than one scanning frozen WSI.

Deep learning model evaluation

The color normalization process for each Whole Slide Image (WSI) during preprocessing was time-consuming, taking up to 10 minutes. To reduce the time spent on data preprocessing, the LGNet's performance was evaluated by comparing the tissue patches that underwent color normalization with those that did not.

Reader study

Previous studies [3-5] have identified several morphological features that suggest PCNSL, including perivascular cuffing of tumor cells, monomorphic nuclei, prominent nucleoli, scant cytoplasm, poorly cohesive cells, the presence of apoptosis, and lack of a fibrillary background. However, some of these features, such as monomorphic nuclei, scant cytoplasm, and lack of a fibrillary background, can also be present in glioma. Additionally, microvascular proliferation and necrosis are more commonly seen in high-grade gliomas. On the other hand, low-grade gliomas are characterized by cytological atypia, which refers to a variation in nuclear shape and size with accompanying hyperchromasia [6]. To evaluate the correlation between these histopathological characteristics and LGNet's misdiagnosis, we included these ten features in our study. Two expert pathologists worked together to identify which

characteristics were present in each slide. We then compared the histopathological features of misdiagnosed cases with those of correctly diagnosed cases to gain a better understanding of LGNet's performance.

Human-machine fusion

Briefly, the fusion prediction p_f for certain slide is defined as a weighted sum of p_m and p_h, where p_m denotes the two-element output probability vector from the deep learning model, and p_h denotes the confidence of the pathologist obtained by a linear transformation from the 6 self-confidence scales [1, 2, 3, 4, 5, 6] to the corresponding probabilities [0.2, 0.32, 0.44, 0.56, 0.68, 0.8], with 0.5 as the threshold of prediction either PCNSL or glioma and either PCNSL or non-PCNSL. Formally, the fused prediction p_f from the deep learning model and the pathologist is defined as below,

$$p_f = \alpha \cdot p_m + (1 - \alpha) \cdot p_h \,, \tag{1}$$

Based on the comparisons between different designs of the weight α in our previous study [7], we finally set α as follow,

$$\alpha = \frac{\frac{1}{u_m}}{\frac{1}{u_m} + \frac{1}{u_h}} \,, \tag{2}$$

where u_m and u_h respectively represent the uncertainties of deep learning model's prediction and the pathologist's diagnosis.

As for the uncertainty of the deep learning model prediction, u_m, the entropy of the two-element output was widely adopted, i.e.,

$$u_m = -\,(p_{m,1} \cdot \ln p_{m,1} + p_{m,2} \cdot \ln p_{m,2}) \,, \tag{3}$$

where $p_{m,1}$ and $p_{m,2}$ are the two elements of deep learning model output p_m, with $p_{m,1}$ representing deep learning model's probability prediction of the input data being glioma or non-PCNSL, and $p_{m,2}$ of being PCNSL and equal to $(1 - p_{m,1})$. The uncertainty of the pathologist's diagnosis, u_h, could be obtained in the same way.

Note that u_m becomes maximum when the model is at the most uncertain status (i.e., $p_{m,1} = p_{m,2} = 0.5$). However, since the threshold *T* to dichotomize the output of the deep learning model has been adjusted to satisfy the EER (equal error rate) requirement on the internal dataset, it suggests that u_m should become maximum when $p_{m,1}$ is equal to the threshold *T*. With this observation, the original $p_{m,1}$ is modified to $p'_{m,1}$ by a simple linear transformation as follows,

$$p'_{m,1} = \begin{cases} p_{m,1}/2T, \ if \ 0<p_{m,1}<T \\ (p_{m,1}+1-2T)/(2-2T), \ if \ T \le p_{m,1} \le 1 \end{cases} \quad (4)$$

and similarly $p_{m,2}$ is modified to $p'_{m,2} = (1 - p'_{m,1})$. The modified $p_{m,1}$ and $p_{m,2}$, called $p'_{m,1}$ and $p'_{m,2}$, were used to estimate the uncertainty u_m based on Formula (3). Also note that the human-machine fusion strategy is predefined and not involved in the training of the deep learning model.

Supplementary results

Deep learning model performance

The LGNet's performance on External Cohort 1 and 2 was evaluated after color normalization during WSI preprocessing. On External Cohort 1, LGNet achieved an AUROC of 0.964 (95% CI 0.94-0.99), sensitivity of 0.918 (95% CI 0.80-0.98), and specificity of 0.849 (95% CI 0.80-0.89). On External Cohort 2, LGNet obtained an AUROC of 0.977 (95% CI 0.95-1.00), sensitivity of 0.955 (95% CI 0.77-1.00), and specificity of 0.824 (95% CI 0.78-0.86) (Supplementary Table 3).

Supplementary Figure 1

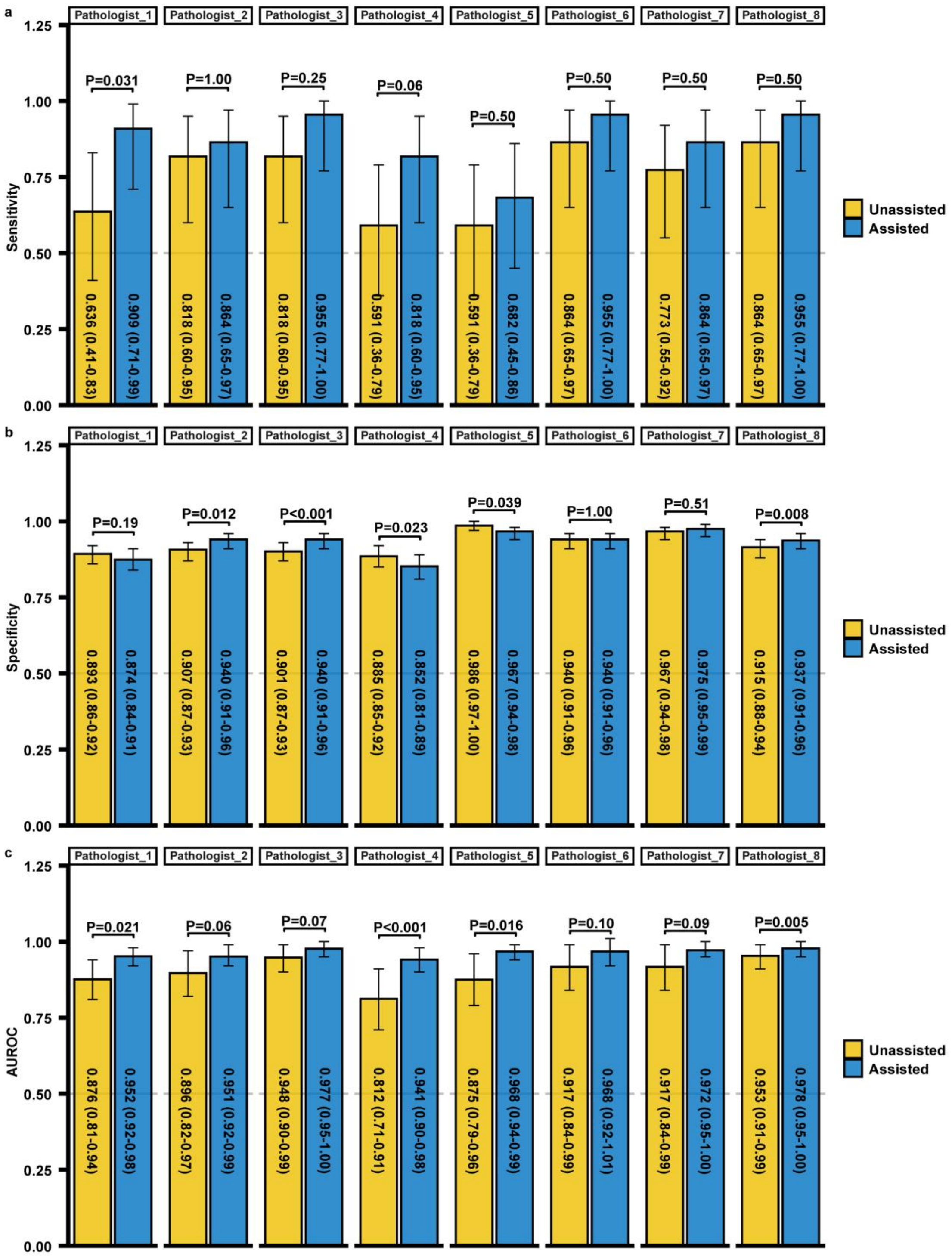

Supplementary Figure 1. The comparison of the diagnostic performance between pathologist with and without the assistance of LGNet on the external cohort 2. The performance was measured using three parameters: Sensitivity (a), Specificity (b), AUROC (c). Pathologist 1 and 4, with one year of experience in intraoperative neuropathological diagnosis; Pathologist 2 and 5, having five years of experience in intraoperative neuropathological diagnosis; Pathologist 3, 6, 7 and 8, having up to ten years of experience in intraoperative neuropathological diagnosis; Pathologist (unassisted), working without the aid of LGNet; Pathologist (assisted), assisted by LGNet; AUROC, the area under the receiver operating characteristic; The error bars are the 95%CI, with the measure of the histogram being the sensitivity, specificity and AUROC of each variable. The sample size to derive statistics is n=386 independent patient samples for each variable. The P value was evaluated from a two-sided test. Adjustments were made for multiple comparisons. The data have been provided in the Source Data file.

Supplementary Figure 2

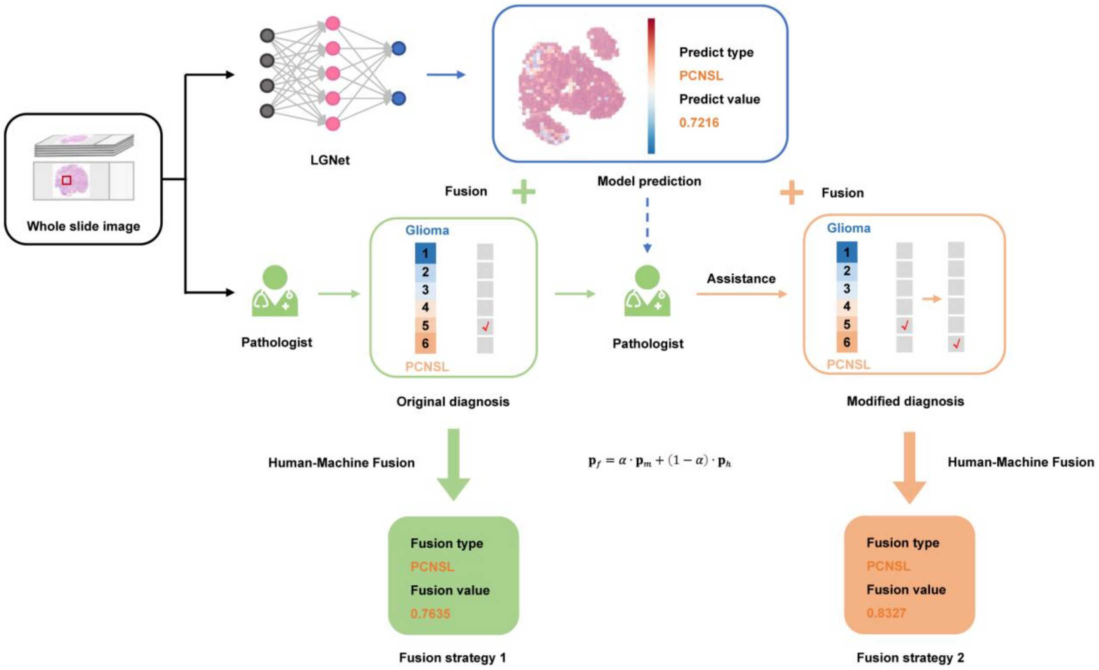

Supplementary Figure 2. The workflow of the human-machine fusion strategies by combining pathologist's diagnosis (original diagnosis or modified diagnosis) with LGNet's prediction. Original diagnosis, the pathologist's diagnosis without the assistance of LGNet; Modified diagnosis, the pathologist's diagnosis with the assistance of LGNet; Fusion strategy 1, the fusion from the LGNet's prediction and pathologist's original diagnosis; Fusion strategy 2, the fusion from the LGNet's prediction and pathologist's modified diagnosis. PCNSL, primary central nervous system lymphoma. Some illustrations were generated with BioRender.com.

Supplementary Figure 3

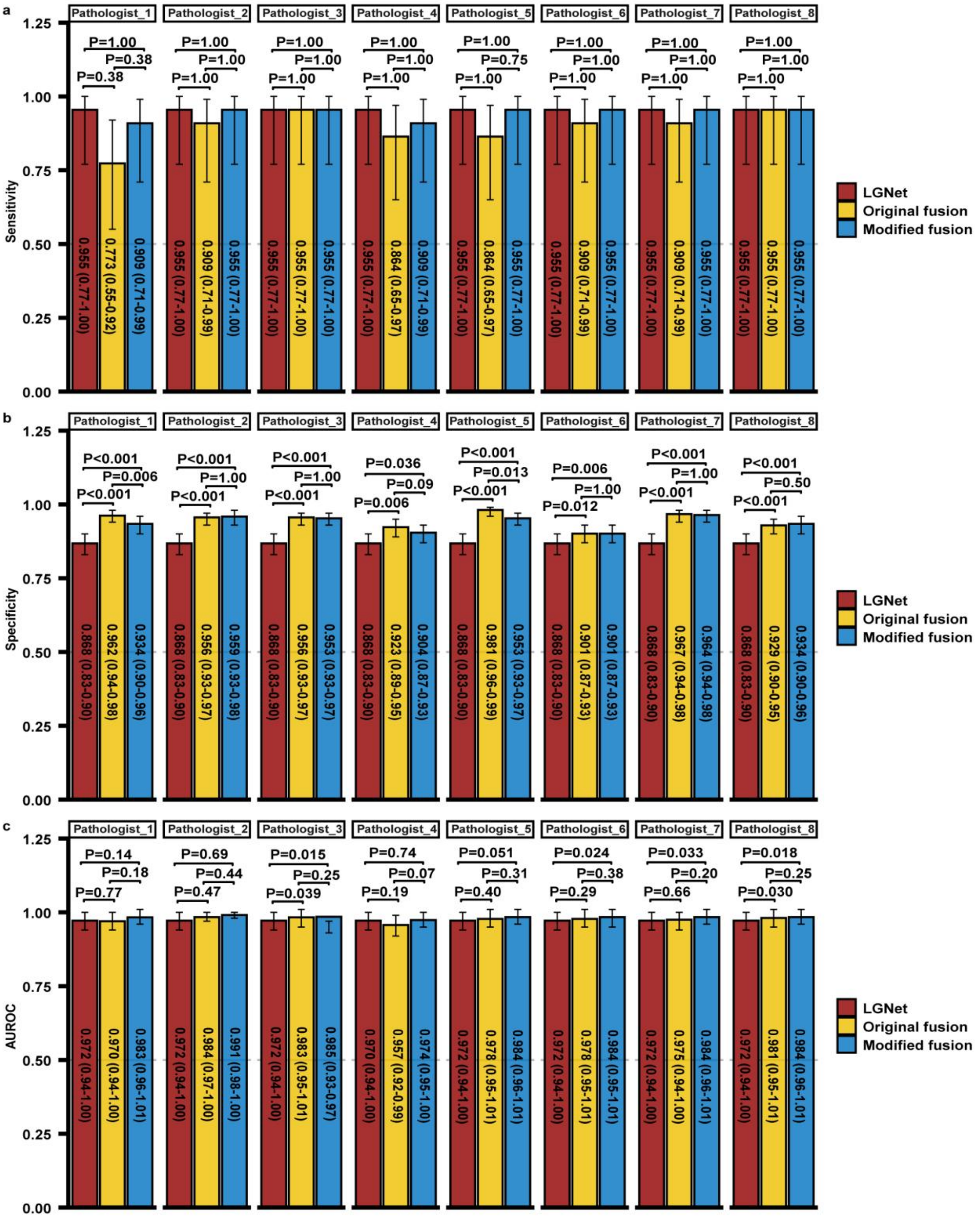

Supplementary Figure 3. The comparison of the fusion prediction from LGNet and pathologist on the external cohort 2. The performance was measured using three parameters: Sensitivity (a), Specificity (b), AUROC (c). Pathologist 1 and 4, with one year of experience in intraoperative neuropathological diagnosis; Pathologist 2 and 5, having five years of experience in intraoperative neuropathological diagnosis; Pathologist 3, 6, 7 and 8, having up to ten years of experience in intraoperative neuropathological diagnosis; Pathologist (unassisted), working without the aid of LGNet; Pathologist (assisted), assisted by LGNet; AUROC, the area under the receiver operating characteristic; Original fusion, the fusion from LGNet's prediction and pathologist's original diagnosis; Modified fusion, the fusion from LGNet's prediction and pathologist's modified diagnosis; The error bars are the 95%CI, with the measure of the histogram being the sensitivity, specificity and AUROC of each variable. The sample size to derive statistics is n=386 independent patient samples for each variable. The P value was evaluated from a two-sided test. Adjustments were made for multiple comparisons. The data have been provided in the Source Data file.

Supplementary Figure 4

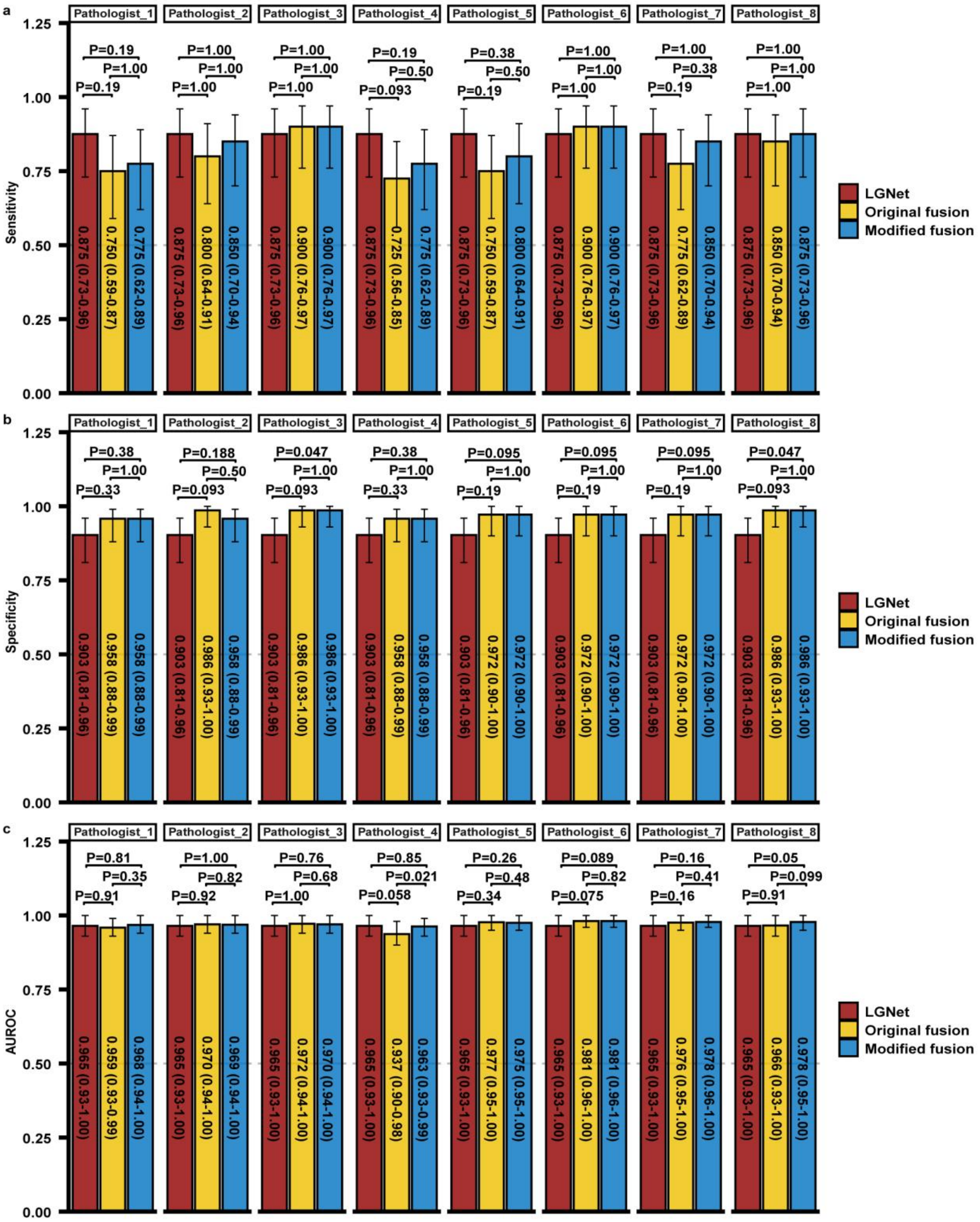

Supplementary Figure 4. The comparison of the fusion prediction from LGNet and pathologist for cases with equivocal imaging diagnosis on the external cohort 1. The performance was measured using three parameters: Sensitivity (a), Specificity (b), AUROC (c). Pathologist 1 and 4, with one year of experience in intraoperative neuropathological diagnosis; Pathologist 2 and 5, having five years of experience in intraoperative neuropathological diagnosis; Pathologist 3, 6, 7 and 8, having up to ten years of experience in intraoperative neuropathological diagnosis; Pathologist (unassisted), working without the aid of LGNet; Pathologist (assisted), assisted by LGNet; AUROC, the area under the receiver operating characteristic; Original fusion, the fusion from LGNet's prediction and pathologist's original diagnosis; Modified fusion, the fusion from LGNet's prediction and pathologist's modified diagnosis; The error bars are the 95%CI, with the measure of the histogram being the sensitivity, specificity and AUROC of each variable. The sample size to derive statistics is n=112 independent patient samples for each variable. The P value was evaluated from a two-sided test. Adjustments were made for multiple comparisons. The data have been provided in the Source Data file.

Supplementary Figure 5

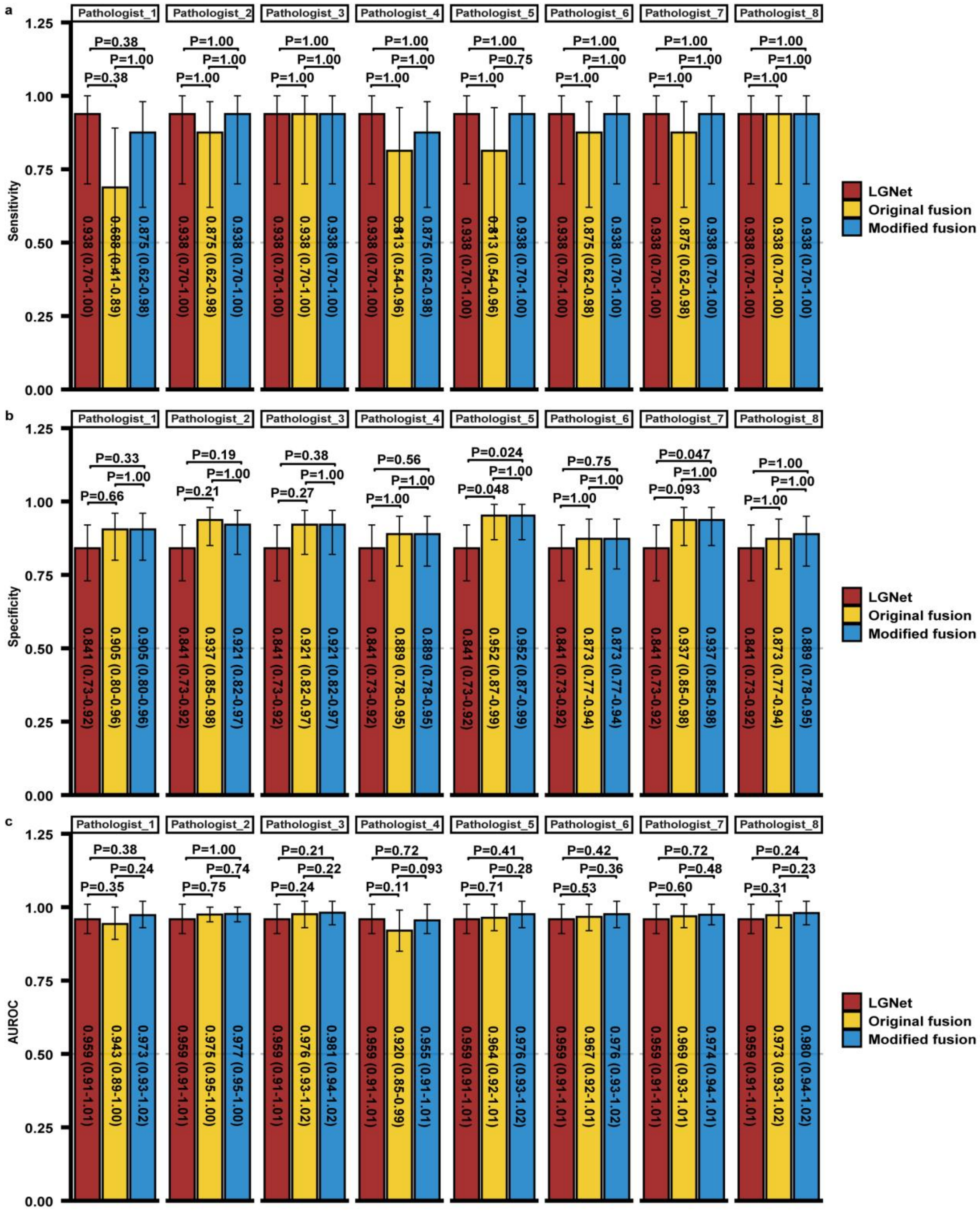

Supplementary Figure 5. The comparison of the fusion prediction from LGNet and pathologist for cases with equivocal imaging diagnosis on the external cohort 2. The performance was measured using three parameters: Sensitivity (a), Specificity (b), AUROC (c). Pathologist 1 and 4, with one year of experience in intraoperative neuropathological diagnosis; Pathologist 2 and 5, having five years of experience in intraoperative neuropathological diagnosis; Pathologist 3, 6, 7 and 8, having up to ten years of experience in intraoperative neuropathological diagnosis; Pathologist (unassisted), working without the aid of LGNet; Pathologist (assisted), assisted by LGNet; AUROC, the area under the receiver operating characteristic; Original fusion, the fusion from LGNet's prediction and pathologist's original diagnosis; Modified fusion, the fusion from LGNet's prediction and pathologist's modified diagnosis; The error bars are the 95%CI, with the measure of the histogram being the sensitivity, specificity and AUROC of each variable. The sample size to derive statistics is n=79 independent patient samples for each variable. The P value was evaluated from a two-sided test. Adjustments were made for multiple comparisons. The data have been provided in the Source Data file.

Supplementary Figure 6

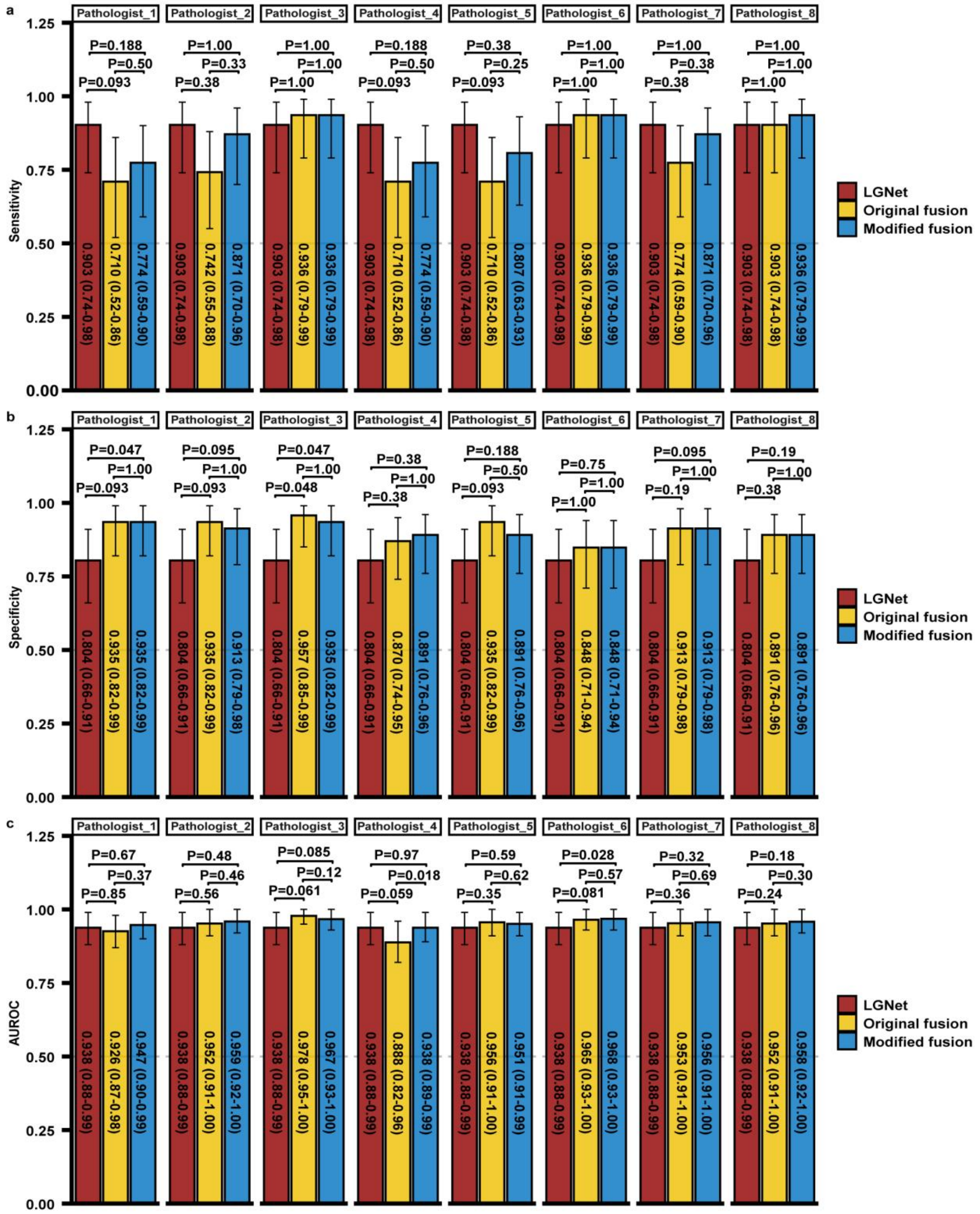

Supplementary Figure 6. The comparison of the fusion prediction from LGNet and pathologist for cases from from the stereotactic biopsy on both external cohorts. The performance was measured using three parameters: Sensitivity (a), Specificity (b), AUROC (c). Pathologist 1 and 4, with one year of experience in intraoperative neuropathological diagnosis; Pathologist 2 and 5, having five years of experience in intraoperative neuropathological diagnosis; Pathologist 3, 6, 7 and 8, having up to ten years of experience in intraoperative neuropathological diagnosis; Pathologist (unassisted), working without the aid of LGNet; Pathologist (assisted), assisted by LGNet; AUROC, the area under the receiver operating characteristic; Original fusion, the fusion from LGNet's prediction and pathologist's original diagnosis; Modified fusion, the fusion from LGNet's prediction and pathologist's modified diagnosis; The error bars are the 95%CI, with the measure of the histogram being the sensitivity, specificity and AUROC of each variable. The sample size to derive statistics is n=76 independent patient samples for each variable. The P value was evaluated from a two-sided test. Adjustments were made for multiple comparisons. The data have been provided in the Source Data file.

Supplementary Figure 7

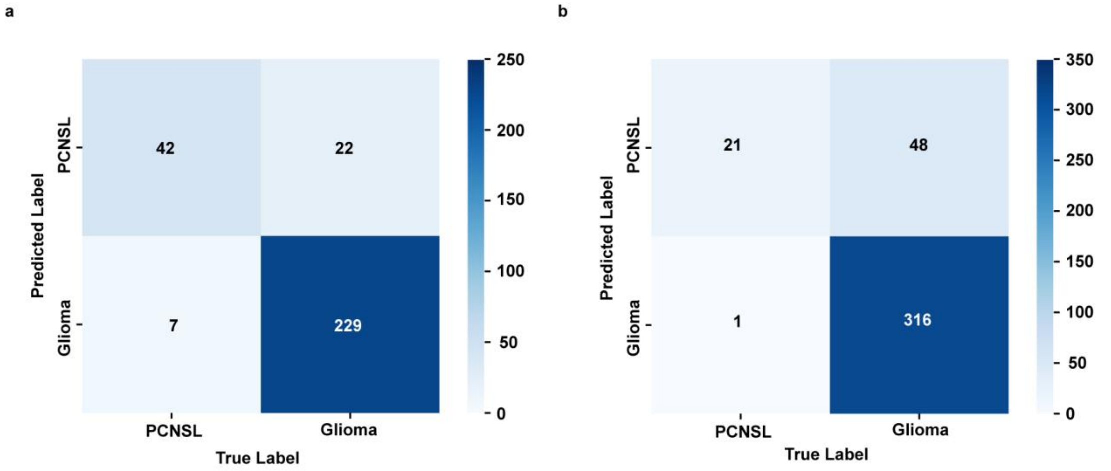

Supplementary Figure 7. Confusion matrix based on LGNet prediction and the ground-truth classification of all patients. a. The confusion matrix of the external cohort 1. b. The confusion matrix of the external cohort 2. The frequencies are shown on a color gradient scale. PCNSL, primary central nervous system lymphoma. The data have been provided in the Source Data file.

Supplementary Figure 8

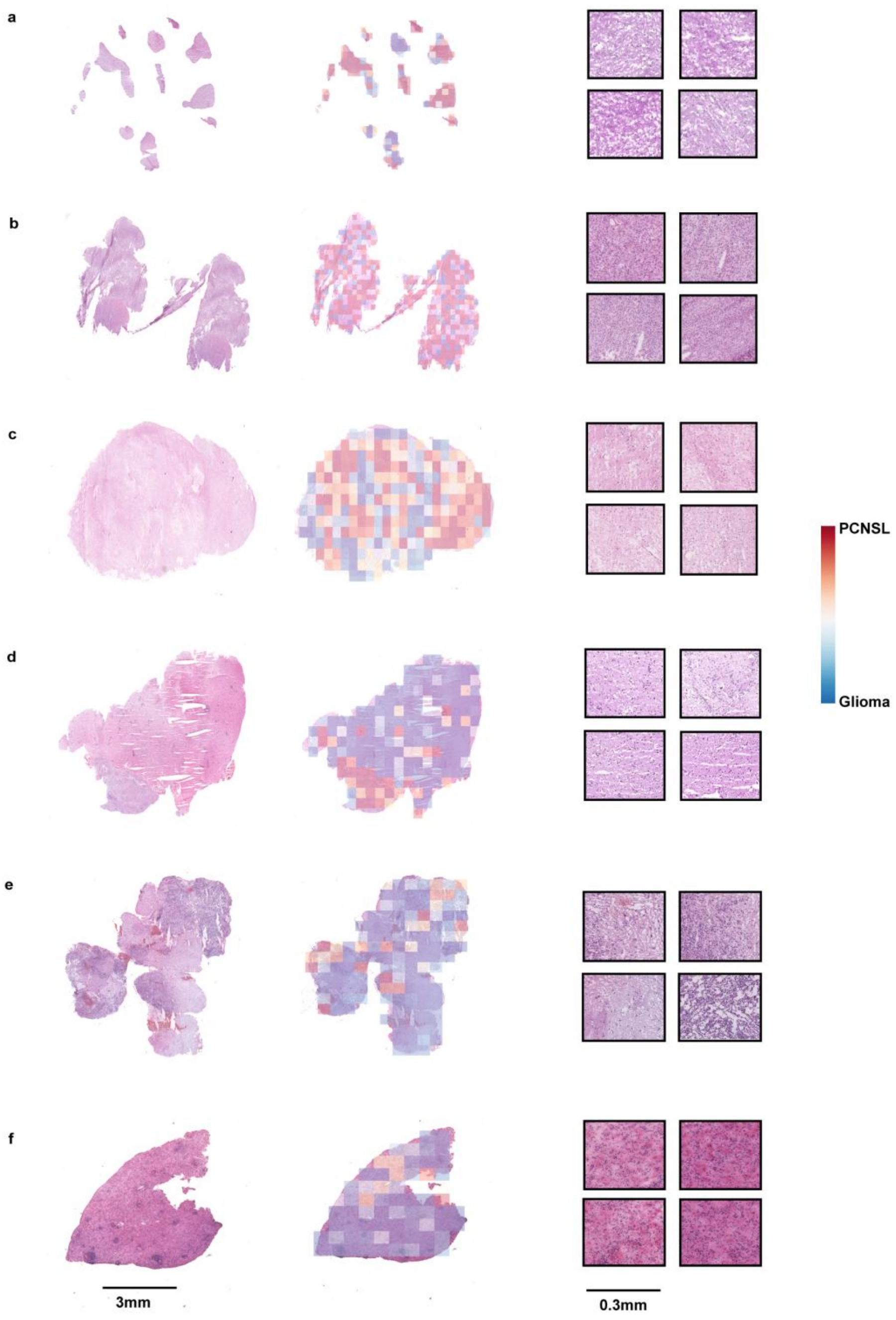

Supplementary Figure 8. The representative cases of unsuccessfully predicted by LGNet. The representative histological images of patients with glioma from the internal cohort (a), External cohort 1 (b), and External cohort 2 (c), respectively. The heatmaps that overlapped on these three WSIs (middle column) revealed that tumor tiles with a high score were mostly predicted as primary central nervous system lymphoma (PCNSL) (reddish color). Tiles with a high score were mainly found in areas with monomorphic nuclei and/or conspicuous nucleoli (right column, tiles magnified 10 times). Internal cohort (d) , External cohort 1 (e) and External cohort 2 (f) histological images (left column) of patients with PCNSL were shown. The overlapping heatmaps on these three WSIs (middle column) revealed that tumor tiles were mostly predicted as glioma with a low score (bluish color). LGNet was able to duplicate all results consistently. Tiles with a low score were more likely to be found in areas with fibrillary background and diversity in nuclear form and size, as well as hyperchromasia (right column, tiles magnified 10 times).

Supplementary Tables

Supplementary Table 1. The baseline of study participants

**Baseline characteristics**	**Internal cohort**	**External cohort 1**	**External cohort 2**	**Proof-of-concept cohort**	***P***
Patients	432	300	386	68	
Histopathological classification					<0.001
Diffuse large B cell lymphoma	79 (18.3%)	49 (16.3%)	22 (5.7%)	7 (10.3%)	
Pilocytic astrocytoma WHO I	22 (5.1%)	20 (6.7%)	30 (7.8%)	3 (4.4%)	
Subependymoma WHO I	1 (0.2%)	1 (0.3%)	1 (0.3%)	0 (0.0%))	
Diffuse astrocytoma WHO II	41 (9.5%)	109 (36.3%)	64 (16.6%)	3 (4.4%)	
Oligodendroglioma WHO II	13 (3.0%)	9 (3.0%)	45 (11.7%)	1 (1.5%)	
Ependymoma WHO II	2 (0.5%)	0 (0.0%)	0 (0.0%)	0 (0.0%)	
Anaplastic astocytoma WHO III	22 (5.1%)	30 (10.0%)	18 (4.7%)	6 (8.8%)	
Anaplastic oligodendroglioma WHO III	29 (6.7%)	8 (2.7%)	26 (6.7%)	2 (2.9%)	
Anaplastic ependymoma WHO III	1 (0.2%)	0 (0.0%)	2 (0.5%)	0 (0.0%)	
Glioblastoma WHO IV	206 (47.7%)	74 (24.7%)	147 (38.1%)	39 (57.4%)	
Diffuse midline glioma WHO IV	16 (3.7%)	0 (0.0%)	31 (8.0%)	7 (10.3%)	
Slides	769	300	386	68	<0.001
PCNSL slides	172 (22.4%)	49 (16.3%)	22 (5.7%)	7 (10.3%)	
Glioma slides	597 (77.6%)	251 (83.7%)	364 (94.3%)	61 (89.7%)	
Age (Mean age, ±SD)	46.558±16.415	42.097±19.668	40.590±17.705	46.529±15.908	<0.001
Gender					0.29
Male	238 (55.1%)	176 (58.7%)	234 (60.6%)	35 (51.5%)	
Female	194 (44.9%)	124 (41.3%)	152 (39.4%)	33 (48.5%)	
Country where patients were recruited					
China	432	300	386	68	
Centers where patients were recruited					<0.001
Sun Yat-sen University Cancer Center	432 (100.0%)	0 (0.0%)	0 (0.0%)	68 (100.0%)	
Zhujiang Hospital,Southern Medical University	0 (0.0%)	300 (100.0%)	0 (0.0%)	0 (0.0%)	
Nanfang Hospital, Southern Medical University	0 (0.0%)	0 (0.0%)	364 (94.3%)	0 (0.0%)	
The First Affiliated Hospital, Sun Yat-sen University	0 (0.0%)	0 (0.0%)	22 (5.7%)	0 (0.0%)	
Race/ethnicity					
Asian	432	300	386	68	
Geographical region					0.10
South of China	323 (74.8%)	235 (78.3%)	307 (79.5%)	46 (67.6%)	
North of China	109 (25.2%)	65 (21.7%)	79 (20.5%)	22 (32.4%)	
Imaging information					<0.001
Suggestive of glioma	254 (58.8%)	176 (58.7%)	303 (78.5%)	38 (55.9%)	
Suggestive of lymphoma	36 (8.3%)	12 (4.0%)	4 (1.0%)	3 (4.4%)	
Equivocal glioma or lymphoma	142 (32.9%)	112 (37.3%)	79 (20.5%)	27 (39.7%)	
Surgery					0.58
Stereotactic biopsy	58 (13.4%)	30 (10.0%)	47 (12.1%)	8 (11.8%)	
Resection biopsy	374 (86.6%)	270 (90.0%)	339 (87.3%)	60 (88.2%)	

Note: The baseline of study participants from different datasets were compared using Chi-square test or variance analysis. The P value was evaluated from a two-sided test. Adjustments were made for multiple comparisons. The data have been provided in the Source Data file.

Supplementary Table 2. The baseline of additional data for internal and two external cohorts

Baseline characteristics	Additional internal dataset	Additional external dataset 1	Additional external dataset 2	*P*
Patients	164	135	80	
Histopathological classification				<0.001
Medulloblastoma	36 (22.0%)	41 (30.4%)	24 (30.0%)	
Central neurocytoma	6 (3.7%)	26 (19.3%)	12 (15.0%)	
Metastatic cancers	99 (60.4%)	51 (37.8%)	24 (30.0%)	
Inflammatory lesions	23 (14.0%)	17 (12.6%)	20 (25.0%)	
Slides	180	135	80	<0.001
Medulloblastoma slides	37 (20.6%)	41 (30.4%)	24 (30.0%)	
Central neurocytoma slides	8 (4.4%)	26 (19.3%)	12 (15.0%)	
Metastatic cancers slides	105(58.3%)	51 (37.8%)	24 (30.0%)	
Inflammatory lesions slides	30 (16.7%)	17 (12.6%)	20 (25.0%)	
Age (Mean age, ±SD)	43.402±22.275	35.356±22.419	35.925±22.424	0.004
Gender				0.53
Male	95 (57.9%)	86 (63.7%)	46 (57.5%)	
Female	69 (42.1%)	49 (36.3%)	34 (42.5%)	

Note: The baseline of study participants from different datasets were compared using Chi-square test or variance analysis. The *P* value was evaluated from a two-sided test. Adjustments were made for multiple comparisons. The data have been provided in the Source Data file.

Supplementary Table 3. The performance of LGNet based on five-fold cross validation on internal cohort

**Slide level**					
Folds	Sensitivity (95% CI)	Specificity (95% CI)	NPV (95% CI)	PPV (95% CI)	AUROC (95% CI)
Fold 1	0.933 (0.68, 1.00)	0.912 (0.81, 0.97)	0.981 (0.90, 1.00)	0.737 (0.49, 0.91)	0.994 (0.98, 1.00)
Fold 2	0.833 (0.59, 0.96)	1.000 (0.94, 1.00)	0.955 (0.87, 0.99)	1.000 (0.78, 1.00)	0.990 (0.97, 1.00)
Fold 3	1.000 (0.77, 1.00)	0.965 (0.88, 1.00)	1.000 (0.94, 1.00)	0.875 (0.62, 0.98)	0.999 (0.99, 1.00)
Fold 4	0.875 (0.68, 0.97)	0.910 (0.82, 0.96)	0.959 (0.89, 0.99)	0.750 (0.55, 0.89)	0.981 (0.96, 1.00)
Fold 5	0.952 (0.76, 1.00)	0.969 (0.89, 1.00)	0.984 (0.92, 1.00)	0.909 (0.71, 0.99)	0.995 (0.99, 1.00)
Averaged	0.919±0.066	0.951±0.039	0.976±0.019	0.854±0.111	0.992±0.007
**Patient level**					
Folds	Sensitivity (95% CI)	Specificity (95% CI)	NPV (95% CI)	PPV (95% CI)	AUROC (95% CI)
Fold 1	1.000 (0.48, 1.00)	0.950 (0.83, 0.99)	1.000 (0.91, 1.00)	0.714 (0.29, 0.96)	1.000 (1.00, 1.00)
Fold 2	0.875 (0.47, 1.00)	1.000 (0.91, 1.00)	0.974 (0.86, 1.00)	1.000 (0.59, 1.00)	0.990 (0.97, 1.01)
Fold 3	1.000 (0.59, 1.00)	1.000 (0.90, 1.00)	1.000 (0.90, 1.00)	1.000 (0.59, 1.00)	1.000 (1.00, 1.00)
Fold 4	0.833 (0.52, 0.98)	1.000 (0.92, 1.00)	0.957 (0.85, 0.99)	1.000 (0.69, 1.00)	1.000 (1.00, 1.00)
Fold 5	1.000 (0.69, 1.00)	0.974 (0.86, 1.00)	1.000 (0.91, 1.00)	0.909 (0.59, 1.00)	0.995 (0.98, 1.00)
Averaged	0.942±0.081	0.985±0.022	0.986±0.198	0.925±0.124	0.997±0.004

Note: 95% CI, 95% confidence intervals; NPV, negative predictive value; PPV, positive predictive value; AUROC, the area under the receiver operating characteristic. The data have been provided in the Source Data file.

Supplementary Table 4. The performance comparison between LGNet with and without color normalization on External cohort 1 and External cohort 2

**Methods**	**External cohort 1**								
	Sensitivity	Difference	*P*	Specificity	Difference	*P*	AUROC	Difference	*P*
LGNet without color normalization (vahadane)	0.857	NA	NA	0.912	NA	NA	0.965	NA	NA
LGNet with color normalization (vahadane)	0.918	0.061	0.25	0.849	-0.063	<0.001	0.964	-0.001	0.75
	**External cohort 2**								
LGNet without color normalization (vahadane)	0.955	NA	NA	0.868	NA	NA	0.972	NA	NA
LGNet with color normalization (vahadane)	0.955	0.000	1.00	0.824	-0.044	0.002	0.977	0.005	0.17

Note: Difference between LGNet with and without color normalization; AUROC, the area under the receiver operating characteristic; NA, not applicable. The difference comparison between AUROCs was used to Delong's test. The McNemar test was used to compare the statistical differences in sensitivity and specificity. The *P* value was evaluated from a two-sided test. Adjustments were made for multiple comparisons. The sample size to derive statistics is n=300 (External cohort 1) and n=386 (External cohort 2) independent patient samples for each variable. The data have been provided in the Source Data file.

Supplementary Table 5. Classification performances of randomly included slides with 8% proportion of PCNSL and glioma on External cohort 2

**Random time**	**AUROC (95% CI)**
Random1	0.970 (0.94, 1.00)
Random2	0.974 (0.94, 1.00)
Random3	0.975 (0.95, 1.00)
Random4	0.971 (0.94, 1.00)
Random5	0.971 (0.94, 1.00)
Random6	0.972 (0.94, 1.00)
Random7	0.973 (0.94, 1.00)
Random8	0.973 (0.94, 1.00)
Random9	0.968 (0.94, 1.00)
Random10	0.976 (0.95, 1.00)
Averaged	0.972

Note: PCNSL, primary central nervous system lymphoma; AUROC, the area under the receiver operating characteristic; 95% CI, 95% confidence intervals.

Supplementary Table 6. The comparison of LGNet's performance and pathologist's performance for cases with equivocal imaging diagnosis on two external cohorts

Cohorts	Category	Predictive performance								
		Sensitivity (95% CI)	P^*	P^a	Specificity (95% CI)	P^*	P^a	AUROC (95% CI)	P^*	P^a
External cohort 1	LGNet	0.875 (0.73, 0.96)	NA	NA	0.903 (0.81, 0.96)	NA	NA	0.965 (0.93, 1.00)	NA	NA
	Pathologist 1	0.525 (0.36, 0.68)	<0.001	0.008	0.931 (0.85, 0.98)	1.00	1.00	0.836 (0.76, 0.91)	<0.001	0.008
	Pathologist 2	0.625 (0.46, 0.77)	0.013	0.05	0.986 (0.93, 1.00)	0.031	0.22	0.877 (0.81, 0.94)	0.008	0.048
	Pathologist 3	0.725 (0.56, 0.85)	0.11	0.22	0.986 (0.93, 1.00)	0.07	0.28	0.923 (0.86, 0.99)	0.15	0.45
	Pathologist 4	0.475 (0.32, 0.64)	<0.001	0.008	0.931 (0.85, 0.98)	0.754	1.00	0.749 (0.65, 0.84)	<0.001	0.008
	Pathologist 5	0.475 (0.32, 0.64)	<0.001	0.008	1.000 (0.95, 1.00)	NA	NA	0.917 (0.87, 0.96)	0.034	0.17
	Pathologist 6	0.750 (0.59, 0.87)	0.18	0.22	0.986 (0.93, 1.00)	0.031	0.22	0.957 (0.93, 0.98)	0.66	0.84
	Pathologist 7	0.600 (0.43, 0.75)	0.001	0.008	0.986 (0.93, 1.00)	0.031	0.22	0.953 (0.92, 0.98)	0.42	0.84
	Pathologist 8	0.700 (0.53, 0.83)	0.039	0.12	0.972 (0.90, 1.00)	0.13	0.39	0.922 (0.87, 0.97)	0.05	0.2
External cohort 2	LGNet	0.938 (0.70, 1.00)	NA	NA	0.841 (0.73, 0.92)	NA	NA	0.959 (0.91, 1.01)	NA	NA
	Pathologist 1	0.563 (0.30, 0.80)	0.031	0.22	0.873 (0.77, 0.94)	0.79	1.00	0.828 (0.72, 0.93)	0.013	0.09
	Pathologist 2	0.875 (0.62, 0.98)	1.00	1.00	0.857 (0.75, 0.93)	1.00	1.00	0.890 (0.80, 0.98)	0.21	0.84
	Pathologist 3	0.813 (0.54, 0.96)	0.50	1.00	0.889 (0.78, 0.95)	0.58	1.00	0.951 (0.90, 1.00)	0.67	1.00
	Pathologist 4	0.500 (0.25, 0.75)	0.016	0.13	0.794 (0.67, 0.89)	0.61	1.00	0.724 (0.58, 0.86)	<0.001	0.008
	Pathologist 5	0.563 (0.30, 0.80)	0.031	0.22	0.984 (0.91, 1.00)	0.012	0.10	0.857 (0.75, 0.96)	0.07	0.42
	Pathologist 6	0.813 (0.54, 0.96)	0.50	1.00	0.905 (0.80, 0.96)	0.22	1.00	0.882 (0.78, 0.98)	0.10	0.50
	Pathologist 7	0.813 (0.54, 0.96)	0.50	1.00	0.921 (0.82, 0.97)	0.18	1.00	0.905 (0.82, 1.00)	0.23	0.84
	Pathologist 8	0.813 (0.54, 0.96)	0.50	1.00	0.952 (0.87, 0.99)	0.065	0.46	0.954 (0.90, 1.00)	0.75	1.00

Note: 95% CI, 95% confidence intervals. The difference comparison between AUROCs was used to Delong's test. The McNemar test was used to compare the statistical differences in sensitivity and specificity. NA, not applicable. The *P* value was evaluated from a two-sided test. Adjustments were made for multiple comparisons. P^a, adjusted P^* value with FDR method. The sample size to derive statistics is n=112 (External cohort 1) and n=79 (External cohort 2) independent patient samples for each variable. The data have been provided in the Source Data file.

Supplementary Table 7. The comparison of LGNet's performance and pathologist's performance for cases from the stereotactic biopsy on both external cohorts

Category	Predictive performance								
	Sensitivity (95% CI)	*P**	P^a	Specificity (95% CI)	*P**	P^a	AUROC (95% CI)	*P**	P^a
LGNet	0.903 (0.74, 0.98)	NA	NA	0.804 (0.66, 0.91)	NA	NA	0.938 (0.88, 0.99)	NA	NA
Pathologist 1	0.516 (0.33, 0.70)	<0.001	0.008	0.804 (0.66, 0.91)	1.00	1.00	0.781 (0.68, 0.89)	0.004	0.028
Pathologist 2	0.710 (0.52, 0.86)	0.07	0.28	0.870 (0.74, 0.95)	0.55	1.00	0.816 (0.72, 0.92)	0.019	0.114
Pathologist 3	0.807 (0.63, 0.93)	0.45	1.00	0.870 (0.75, 0.95)	0.58	1.00	0.947 (0.90, 0.99)	0.77	1.00
Pathologist 4	0.419 (0.25, 0.61)	<0.001	0.008	0.826 (0.69, 0.92)	1.000	1.00	0.648 (0.52, 0.78)	<0.001	0.008
Pathologist 5	0.419 (0.25, 0.61)	<0.001	0.008	1.000 (0.92, 1.00)	NA	NA	0.842 (0.76, 0.93)	0.031	0.155
Pathologist 6	0.871 (0.70, 0.96)	1.00	1.00	0.957 (0.85, 0.99)	0.016	0.11	0.953 (0.91, 1.00)	0.66	1.00
Pathologist 7	0.645 (0.45, 0.81)	0.008	0.040	0.935 (0.82, 0.99)	0.11	0.66	0.861 (0.78, 0.94)	0.07	0.28
Pathologist 8	0.839 (0.66, 0.95)	0.63	1.00	0.870 (0.74, 0.95)	0.45	1.00	0.930 (0.88, 0.98)	0.73	1.00

Note: 95% CI, 95% confidence intervals. The difference comparison between AUROCs was used to Delong's test. The McNemar test was used to compare the statistical differences in sensitivity and specificity. NA, not applicable. The *P* value was evaluated from a two-sided test. Adjustments were made for multiple comparisons. P^a, adjusted *P** value with FDR method. The sample size to derive statistics is n=76 independent patient samples for each variable. The data have been provided in the Source Data file.

Supplementary Table 8. The comparison of the diagnostic performance between the pathologist, human-machine fusion and the model in distinguishing PCNSL from non-PCNSL on the external test dataset 1 and 2 including PCNSL and non-PCNSL

Dataset	Category	Predictive performance								
		Sensitivity (95% CI)	P*	P^a	Specificity (95% CI)	P*	P^a	AUROC (95% CI)	P*	P^a
The external test dataset 1 including PCNSL and non-PCNSL	Model	0.980 (0.89, 1.00)	NA	NA	0.847 (0.81, 0.88)	NA	NA	0.981 (0.97, 0.99)	NA	NA
	Pathologist 3	0.735 (0.59, 0.85)	0.002	0.006	0.946 (0.92, 0.97)	<0.001	0.003	0.912 (0.86, 0.97)	0.012	0.036
	Original fusion	0.939 (0.83, 0.99)	0.63	1.00	0.959 (0.93, 0.98)	<0.001	0.003	0.988 (0.98, 1.00)	0.25	0.32
	Modified fusion	0.939 (0.83, 0.99)	0.63	1.00	0.961 (0.94, 0.98)	<0.001	0.003	0.989 (0.98, 1.00)	0.16	0.32
The external test dataset 2 including PCNSL and non-PCNSL	Model	1.000 (0.85, 1.00)	NA	NA	0.800 (0.76, 0.84)	NA	NA	0.993 (0.99, 1.00)	NA	NA
	Pathologist 3	0.818 (0.60, 0.95)	NA	NA	0.890 (0.86, 0.92)	<0.001	0.003	0.940 (0.89, 0.99)	0.043	0.11
	Original fusion	0.956 (0.77, 1.00)	NA	NA	0.930 (0.90, 0.95)	<0.001	0.003	0.993 (0.98, 1.00)	0.83	0.83
	Modified fusion	1.000 (0.85, 1.00)	NA	NA	0.941 (0.92, 0.96)	<0.001	0.003	0.998 (0.99, 1.00)	0.037	0.11

Note: 95% CI, 95% confidence intervals; Model, the model in distinguishing PCNSL from non-PCNSL; Original fusion, the fusion from Model's prediction and pathologist's original diagnosis; Modified fusion, the fusion from Model's prediction and pathologist's modified diagnosis; The difference comparison between AUROCs was used to Delong's test. The McNemar test was used to compare the statistical differences in sensitivity and specificity. NA, not applicable. The P value was evaluated from a two-sided test. Adjustments were made for multiple comparisons. P^a, adjusted P* value with FDR method. The sample size to derive statistics is n=435 (The external test dataset 1) and n=466 (The external test dataset 2) independent patient samples for each variable. The data have been provided in the Source Data file.

Supplementary Table 9. The comparison of the diagnostic performance between the pathologist without and with the aid of LGNet for cases with equivocal imaging diagnosis on External cohort 1

Category	Predictive performance					
	Sensitivity (95% CI)	*P**	Specificity (95% CI)	*P**	AUROC (95% CI)	*P**
Pathologist 1 (Unassisted)	0.525 (0.36, 0.68)	0.016	0.931 (0.85, 0.98)	0.38	0.836 (0.76, 0.91)	0.004
Pathologist 1 (Assisted)	0.700 (0.53, 0.83)		0.958 (0.88, 0.99)		0.893 (0.83, 0.96)	
Pathologist 2 (Unassisted)	0.625 (0.46, 0.77)	0.021	0.986 (0.93, 1.00)	0.50	0.877 (0.81, 0.94)	0.013
Pathologist 2 (Assisted)	0.825 (0.67, 0.93)		0.958 (0.88, 0.99)		0.934 (0.88, 0.99)	
Pathologist 3 (Unassisted)	0.725 (0.56, 0.85)	0.031	0.986 (0.93, 1.00)	1.00	0.923 (0.86, 0.99)	0.012
Pathologist 3 (Assisted)	0.875 (0.73, 0.96)		0.986 (0.93, 1.00)		0.943 (0.89, 1.00)	
Pathologist 4 (Unassisted)	0.475 (0.32, 0.64)	0.008	0.931 (0.85, 0.98)	0.63	0.749 (0.65, 0.84)	<0.001
Pathologist 4 (Assisted)	0.675 (0.51, 0.81)		0.958 (0.88, 0.99)		0.888 (0.82, 0.96)	
Pathologist 5 (Unassisted)	0.475 (0.32, 0.64)	0.002	1.000 (0.95, 1.00)	NA	0.917 (0.87, 0.96)	0.12
Pathologist 5 (Assisted)	0.725 (0.56, 0.85)		0.986 (0.93, 1.00)		0.949 (0.91, 0.99)	
Pathologist 6 (Unassisted)	0.750 (0.59, 0.87)	0.06	0.986 (0.93, 1.00)	1.00	0.957 (0.93, 0.98)	0.010
Pathologist 6 (Assisted)	0.875 (0.73, 0.96)		0.972 (0.90, 1.00)		0.988 (0.97, 1.00)	
Pathologist 7 (Unassisted)	0.600 (0.43, 0.75)	0.031	0.986 (0.93, 1.00)	1.00	0.953 (0.92, 0.98)	0.016
Pathologist 7 (Assisted)	0.750 (0.59, 0.87)		0.986 (0.93, 1.00)		0.976 (0.95, 1.00)	
Pathologist 8 (Unassisted)	0.700 (0.53, 0.83)	0.031	0.972 (0.90, 1.00)	1.00	0.922 (0.87, 0.97)	0.001
Pathologist 8 (Assisted)	0.850 (0.70, 0.94)		0.986 (0.93, 1.00)		0.965 (0.94, 1.00)	

Note: 95% CI, 95% confidence intervals. The difference comparison between AUROCs was used to Delong's test. The McNemar test was used to compare the statistical differences in sensitivity and specificity. NA, not applicable. The *P* value was evaluated from a two-sided test. Adjustments were made for multiple comparisons. The sample size to derive statistics is n=112 independent patient samples for each variable. The data have been provided in the Source Data file.

Supplementary Table 10. The comparison of the diagnostic performance between the pathologist without and with the aid of LGNet for cases with equivocal imaging diagnosis on External cohort 2

**Category**	**Predictive performance**					
	Sensitivity (95% CI)	*P**	Specificity (95% CI)	*P**	AUROC (95% CI)	*P**
Pathologist 1 (Unassisted)	0.563 (0.30, 0.80)	0.063	0.873 (0.77, 0.94)	0.25	0.828 (0.72, 0.93)	0.019
Pathologist 1 (Assisted)	0.875 (0.62, 0.98)		0.825 (0.71, 0.91)		0.941 (0.89, 0.99)	
Pathologist 2 (Unassisted)	0.875 (0.62, 0.98)	1.00	0.857 (0.75, 0.93)	0.63	0.890 (0.80, 0.98)	0.23
Pathologist 2 (Assisted)	0.875 (0.62, 0.98)		0.889 (0.78, 0.95)		0.932 (0.87, 0.99)	
Pathologist 3 (Unassisted)	0.813 (0.54, 0.96)	0.50	0.889 (0.78, 0.95)	0.13	0.951 (0.90, 1.00)	0.035
Pathologist 3 (Assisted)	0.938 (0.70, 1.00)		0.952 (0.87, 0.99)		0.976 (0.94, 1.01)	
Pathologist 4 (Unassisted)	0.500 (0.25, 0.75)	0.13	0.794 (0.67, 0.89)	1.00	0.724 (0.58, 0.86)	<0.001
Pathologist 4 (Assisted)	0.750 (0.48, 0.93)		0.778 (0.66, 0.87)		0.907 (0.83, 0.98)	
Pathologist 5 (Unassisted)	0.563 (0.30, 0.80)	0.50	0.984 (0.91, 1.00)	0.50	0.857 (0.75, 0.96)	0.05
Pathologist 5 (Assisted)	0.688 (0.41, 0.89)		0.952 (0.87, 0.99)		0.955 (0.91, 1.00)	
Pathologist 6 (Unassisted)	0.813 (0.54, 0.96)	0.50	0.905 (0.80, 0.96)	1.00	0.882 (0.78, 0.98)	0.10
Pathologist 6 (Assisted)	0.938 (0.70, 1.00)		0.905 (0.80, 0.96)		0.949 (0.89, 1.01)	
Pathologist 7 (Unassisted)	0.813 (0.54, 0.96)	1.00	0.921 (0.82, 0.97)	0.50	0.905 (0.82, 1.00)	0.17
Pathologist 7 (Assisted)	0.875 (0.62, 0.98)		0.952 (0.87, 0.99)		0.960 (0.92, 1.00)	
Pathologist 8 (Unassisted)	0.813 (0.54, 0.96)	0.50	0.952 (0.87, 0.99)	1.00	0.954 (0.90, 1.00)	0.034
Pathologist 8 (Assisted)	0.938 (0.70, 1.00)		0.968 (0.89, 1.00)		0.978 (0.94, 1.02)	

Note: 95% CI, 95% confidence intervals. The difference comparison between AUROCs was used to Delong's test. The McNemar test was used to compare the statistical differences in sensitivity and specificity.The *P* value was evaluated from a two-sided test. Adjustments were made for multiple comparisons. The sample size to derive statistics is n=79 independent patient samples for each variable. The data have been provided in the Source Data file.

Supplementary Table 11. The comparison of the diagnostic performance between the pathologist without and with the aid of LGNet for cases from the stereotactic biopsy on both external cohorts

Category	Predictive performance					
	Sensitivity (95% CI)	*P*	Specificity (95% CI)	*P*	AUROC (95% CI)	*P*
Pathologist 1 (Unassisted)	0.516 (0.33, 0.70)	0.016	0.804 (0.66, 0.91)	0.38	0.781 (0.68, 0.89)	0.006
Pathologist 1 (Assisted)	0.742 (0.55, 0.88)		0.870 (0.74, 0.95)		0.884 (0.81, 0.96)	
Pathologist 2 (Unassisted)	0.710 (0.52, 0.86)	0.13	0.870 (0.74, 0.95)	1.00	0.816 (0.72, 0.92)	0.038
Pathologist 2 (Assisted)	0.839 (0.66, 0.95)		0.848 (0.71, 0.94)		0.885 (0.81, 0.96)	
Pathologist 3 (Unassisted)	0.807 (0.63, 0.93)	0.13	0.870 (0.75, 0.95)	0.38	0.947 (0.90, 0.99)	0.36
Pathologist 3 (Assisted)	0.936 (0.79, 0.99)		0.935 (0.82, 0.99)		0.959 (0.92, 1.00)	
Pathologist 4 (Unassisted)	0.419 (0.25, 0.61)	0.008	0.826 (0.69, 0.92)	0.63	0.648 (0.52, 0.78)	<0.001
Pathologist 4 (Assisted)	0.677 (0.49, 0.83)		0.870 (0.74, 0.95)		0.857 (0.77, 0.94)	
Pathologist 5 (Unassisted)	0.419 (0.25, 0.61)	0.031	1.000 (0.92, 1.00)	NA	0.842 (0.76, 0.93)	0.007
Pathologist 5 (Assisted)	0.613 (0.42, 0.78)		0.957 (0.85, 0.99)		0.940 (0.90, 0.99)	
Pathologist 6 (Unassisted)	0.871 (0.70, 0.96)	0.25	0.957 (0.85, 0.99)	1.00	0.953 (0.91, 1.00)	0.16
Pathologist 6 (Assisted)	0.968 (0.83, 1.00)		0.935 (0.82, 0.99)		0.981 (0.96, 1.00)	
Pathologist 7 (Unassisted)	0.645 (0.45, 0.81)	0.25	0.935 (0.82, 0.99)	1.00	0.861 (0.78, 0.94)	0.011
Pathologist 7 (Assisted)	0.742 (0.55, 0.88)		0.935 (0.82, 0.99)		0.936 (0.89, 0.98)	
Pathologist 8 (Unassisted)	0.839 (0.66, 0.95)	0.25	0.870 (0.74, 0.95)	1.00	0.930 (0.88, 0.98)	0.043
Pathologist 8 (Assisted)	0.936 (0.79, 0.99)		0.891 (0.76, 0.96)		0.957 (0.91, 1.00)	

Note: 95% CI, 95% confidence intervals. The difference comparison between AUROCs was used to Delong's test. The McNemar test was used to compare the statistical differences in sensitivity and specificity. NA, not applicable. The *P* value was evaluated from a two-sided test. Adjustments were made for multiple comparisons. The sample size to derive statistics is n=76 independent patient samples for each variable. The data have been provided in the Source Data file.

Supplementary Table 12. The summary of histomorphological features of misdiagnosed cases by LGNet on External cohort 1 and 2

Clinicopathological features		External cohort 1			External cohort 2		
		Glioma misdiagnosed as PCNSL (n=22)	Correctly diagnosed glioma (n=229)	*P*	Glioma misdiagnosed as PCNSL (n=48)	Correctly diagnosed glioma (n=316)	*P*
Perivascular cuffing of tumor cells	Presence	0 (0.0%)	0 (0.0%)	NA	0 (0.0%)	0 (0.0%)	NA
	Absence	22 (100.0%)	229 (100.0%)		48 (100.0%)	316 (100.0%)	
Monomorphic nuclei	Presence	4 (18.2%)	6 (2.9%)	<0.001	2 (4.2%)	17 (5.4%)	1.000
	Absence	18 (81.8%)	223 (97.1%)		46 (95.8%)	299 (94.6%)	
Prominent nucleoli	Presence	3 (13.6%)	2 (0.9%)	<0.001	0 (0.0%)	8 (2.5%)	0.604
	Absence	19 (86.4%)	227 (99.1%)		48 (100.0%)	308 (97.5%)	
Scant cytoplasm	Presence	5 (22.7%)	36 (15.7%)	0.396	7 (14.6%)	58 (18.4%)	0.525
	Absence	17 (77.3%)	193 (84.3%)		41 (85.4%)	258 (81.6%)	
Poorly cohesive	Presence	5 (22.7%)	23 (10.0%)	0.071	1 (2.1%)	8 (2.5%)	1.000
	Absence	17 (77.3%)	206 (90.0%)		47 (97.9%)	308 (97.5%)	
Apoptosis	Presence	0 (0.0%)	0 (0.0%)	NA	0 (0.0%)	0 (0.0%)	NA
	Absence	22 (100.0%)	229(100.0%)		48 (100.0%)	316 (100.0%)	
Fibrillary background	Presence	19 (86.4%)	194 (84.7%)	0.837	43 (89.6%)	274 (86.7%)	0.747
	Absence	3 (13.6%)	35 (15.3%)		5 (10.4%)	42 (13.3%)	
Variation in nuclear shape and size with accompanying hyperchromasia	Presence	19 (86.4%)	223 (97.4%)	0.008	47 (97.9%)	297 (94.0%)	0.493
	Absence	3 (13.6%)	6 (2.6%)		1 (2.1%)	19 (6.0%)	
Microvascular proliferation	Presence	2 (9.1%)	34 (14.8%)	0.462	9 (18.8%)	104 (32.9%)	0.048
	Absence	20 (90.9%)	195 (85.2%)		39 (81.2%)	212 (67.1%)	
Necrosis	Presence	1 (4.5%)	20 (8.7%)	0.498	3 (6.2%)	41 (13.0%)	0.274
	Absence	21 (95.5%)	209 (91.3%)		45 (93.8%)	275 (87.0%)	

Note: PCNSL, primary central nervous system lymphoma. The features of misdiagnosed cases were compared with those of correctly diagnosed cases with the Chi-square test. NA, not applicable. The *P* value was evaluated from a two-sided test. Adjustments were made for multiple comparisons. The sample size to derive statistics is n=300 (External cohort 1) and n=386 (External cohort 2) independent patient samples for each variable. The data have been provided in the Source Data file.

Supplementary Table 13. The comparison of the diagnostic performance between the pathologist without and with the aid of LGNet on the proof-of-concept cohort

**Performances**	**Pathologist A (unassisted)**	**Pathologist A (assisted)**	***P****	**Pathologist B (unassisted)**	**Pathologist B (assisted)**	***P****
Sensitivity (95% CI)	0.571 (0.18,0.90)	1.000 (0.59,1.00)	1.00	0.857 (0.42,1.00)	1.000 (0.59,1.00)	NA
Specificity (95% CI)	0.836 (0.72,0.92)	0.967 (0.89,1.00)	1.00	0.967 (0.89,1.00)	0.967 (0.89,1.00)	1.00
AUROC (95% CI)	0.821 (0.70,0.94)	0.991 (0.98,1.01)	0.003	0.972 (0.92,1.02)	0.991 (0.97,1.01)	0.26

Note: 95% CI, 95% confidence intervals. * indicates the comparison of the difference between each pathologist both without and with assistance; Pathologist A, the pathologist with one year of experience in intraoperative diagnosis; Pathologist B, the pathologist with up to 10 years of experience in intraoperative diagnosis; Pathologist A (unassisted): Pathologist A without the assistance of LGNet; Pathologist A (assisted): Pathologist A with the assistance of LGNet; Pathologist B (unassisted): Pathologist B without the assistance of LGNet; Pathologist B (assisted): Pathologist B with the assistance of LGNet; AUROC, the area under the receiver operating characteristic; NA, not applicable. The difference comparison between AUROCs was used to Delong's test. The McNemar test was used to compare the statistical differences in sensitivity and specificity. The *P* value was evaluated from a two-sided test. Adjustments were made for multiple comparisons. The sample size to derive statistics is n=68 independent patient samples for each variable. The data have been provided in the Source Data file.

Supplementary References

1. Otsu, N. A Threshold Selection Method from Gray-Level Histograms. *IEEE Transactions on Systems, Man, and Cybernetics* **9**, 62-66 (1979).
2. Vahadane, A.*, et al.* Structure-Preserving Color Normalization and Sparse Stain Separation for Histological Images. *IEEE Trans Med Imaging* **35**, 1962-1971 (2016).
3. Sugita, Y.*, et al.* Intraoperative rapid diagnosis of primary central nervous system lymphomas: advantages and pitfalls. *Neuropathology* **34**, 438-445 (2014).
4. Hattab, E.M.*, et al.* Most primary central nervous system diffuse large B-cell lymphomas occurring in immunocompetent individuals belong to the nongerminal center subtype: a retrospective analysis of 31 cases. *Mod Pathol* **23**, 235-243 (2010).
5. Cha, Y.J., Choi, J. & Kim, S.H. Presence of apoptosis distinguishes primary central nervous system lymphoma from glioblastoma during intraoperative consultation. *Clin Neuropathol* **37**, 105-111 (2018).
6. Louis, D.N.*, et al.* The 2007 WHO classification of tumours of the central nervous system. *Acta Neuropathol* **114**, 97-109 (2007).
7. Zheng, X.*, et al.* A deep learning model and human-machine fusion for prediction of EBV-associated gastric cancer from histopathology. *Nat Commun* **13**, 2790 (2022).
