# Supplementary material for: A multicenter proof-of-concept study on deep learning-based intraoperative discrimination of primary central nervous system lymphoma
Source: Nat Commun. 2024 May 4;15:3768. doi: 10.1038/s41467-024-48171-x (PMC11069536; doi:10.1038/s41467-024-48171-x)
Supplement: Supplementary file 3 — Reporting Summary [file 41467_2024_48171_MOESM3_ESM.pdf]

nature portfolio

Corresponding author(s): Mu-Yan Cai

Last updated by author(s): 2024-3-14

# Reporting Summary

Nature Portfolio wishes to improve the reproducibility of the work that we publish. This form provides structure for consistency and transparency in reporting. For further information on Nature Portfolio policies, see our Editorial Policies and the Editorial Policy Checklist.

## Statistics

For all statistical analyses, confirm that the following items are present in the figure legend, table legend, main text, or Methods section.

| n/a | Confirmed | |
|---|---|---|
| ☐ | ☒ | The exact sample size ($n$) for each experimental group/condition, given as a discrete number and unit of measurement |
| ☐ | ☒ | A statement on whether measurements were taken from distinct samples or whether the same sample was measured repeatedly |
| ☐ | ☒ | The statistical test(s) used AND whether they are one- or two-sided<br>*Only common tests should be described solely by name; describe more complex techniques in the Methods section.* |
| ☒ | ☐ | A description of all covariates tested |
| ☐ | ☒ | A description of any assumptions or corrections, such as tests of normality and adjustment for multiple comparisons |
| ☐ | ☒ | A full description of the statistical parameters including central tendency (e.g. means) or other basic estimates (e.g. regression coefficient) AND variation (e.g. standard deviation) or associated estimates of uncertainty (e.g. confidence intervals) |
| ☐ | ☒ | For null hypothesis testing, the test statistic (e.g. $F$, $t$, $r$) with confidence intervals, effect sizes, degrees of freedom and $P$ value noted<br>*Give P values as exact values whenever suitable.* |
| ☒ | ☐ | For Bayesian analysis, information on the choice of priors and Markov chain Monte Carlo settings |
| ☒ | ☐ | For hierarchical and complex designs, identification of the appropriate level for tests and full reporting of outcomes |
| ☒ | ☐ | Estimates of effect sizes (e.g. Cohen's $d$, Pearson's $r$), indicating how they were calculated |

*Our web collection on statistics for biologists contains articles on many of the points above.*

## Software and code

Policy information about availability of computer code

| | |
|---|---|
| Data collection | The data was collected from our local medical center and multiple medical centers , which were described in the supplementary methods section. |
| Data analysis | The data were processed with the source code which was available to editors and reviewers. The source code can be available along the publication of the paper. |

For manuscripts utilizing custom algorithms or software that are central to the research but not yet described in published literature, software must be made available to editors and reviewers. We strongly encourage code deposition in a community repository (e.g. GitHub). See the Nature Portfolio guidelines for submitting code & software for further information.

## Data

Policy information about availability of data

All manuscripts must include a data availability statement. This statement should provide the following information, where applicable:

- Accession codes, unique identifiers, or web links for publicly available datasets
- A description of any restrictions on data availability
- For clinical datasets or third party data, please ensure that the statement adheres to our policy

The data were processed with the source code which was available to editors and reviewers. The source code can be available along the publication of the paper.

## Research involving human participants, their data, or biological material

Policy information about studies with human participants or human data. See also policy information about sex, gender (identity/presentation), and sexual orientation and race, ethnicity and racism.

| | |
|---|---|
| Reporting on sex and gender | The internal cohort comprised 238 males and 194 females. External cohort 1 included 176 males and 124 females, while External cohort 2 consisted of 234 males and 152 females. The proof of concept study cohort had 35 males and 33 females. The additional internal dataset, external dataset 1 and 2 included 95 males and 69 females, 86 males and 49 females, and 46 males and 34 females.The gender of participants was considered in the study design and reported in the characteristics of different cohorts, and determined on self-report. A specific gender-based analysis a priori was not performed. |
| Reporting on race, ethnicity, or other socially relevant groupings | The race/ethnicity of all patients were Asian and were not involved socially constructed or socially relevant categorization variables in our study. |
| Population characteristics | To develop and validate the LGNet, all patients in our study were diagnosed as either PCNSL or glioma. Patients from Internal cohort had an average age of 46.6 years. The average age of patients with External cohort 1 and 2 was 42.1 and 40.6 years, respectively. The proof-of-concept study cohort patients had an average age of 46.5 years. In addition, to further expand and validate the deep learning model's capacity to differentiate PCNSL from non-PCNSL (including glioma and other brain lesions), additional internal dataset, external dataset 1 and 2 was collected, the average age of patients was 43.4, 35.3, and 36.2, respectively. |
| Recruitment | Patients were not directly involved or recruited for the study. This study involved retrospective analysis of pathology slides from patients obtained during standard clinical care. |
| Ethics oversight | The IRB committee of Sun Yat-sen University Cancer Center approved the study. Only retrospective data were used for the research, without any active patient involvement. |

Note that full information on the approval of the study protocol must also be provided in the manuscript.

# Field-specific reporting

Please select the one below that is the best fit for your research. If you are not sure, read the appropriate sections before making your selection.

☒ Life sciences ☐ Behavioural & social sciences ☐ Ecological, evolutionary & environmental sciences

For a reference copy of the document with all sections, see nature.com/documents/nr-reporting-summary-flat.pdf

# Life sciences study design

All studies must disclose on these points even when the disclosure is negative.

| | |
|---|---|
| Sample size | No sample size calculations were performed. To develop and validate the LGNet, we used all available slides (172 slides) from patients with PCNSL and glioma slides (597 slides) from the pool of all patients with glioma from the internal dataset for model development and held out testing. All available slides from External cohort1 (300 slides ) and 2 (386 slides ) and the proof-of-concept cohort (68 slides) were used for additional testing. In addition, to further expand and validate the deep learning model's capacity to differentiate PCNSL from non-PCNSL (including glioma and other brain lesions), we broadened our dataset (internal cohort, external cohort 1 and 2) to encompass frozen section images of various brain lesions like smedulloblastoma, central neurocytoma, metastatic cancer, and inflammation lesions. |
| Data exclusions | The exclusion criteria included the followings: (1) unqualified slide scanning (such as slides out of focus, dull staining or having large folds), and (2) no available H&E-stained frozen samples. |
| Replication | Code for our training and evaluation protocols will be provided for reproducibility. Attempts at replication were successful for all results reported in the study. |
| Randomization | During training, the internal dataset was randomly divided into five folds at the slide level, and the five-fold cross-validation strategy was employed to train five classifiers. |
| Blinding | Blinded to all clinical information and the performance of deep learning model, pathologists reviewed these slides and classified each case into PCNSL or glioma and other brain lesions based on their expertise and experience. |

# Reporting for specific materials, systems and methods

We require information from authors about some types of materials, experimental systems and methods used in many studies. Here, indicate whether each material, system or method listed is relevant to your study. If you are not sure if a list item applies to your research, read the appropriate section before selecting a response.

## Materials & experimental systems

| n/a | Involved in the study |
| --- | --- |
| ☒ | ☐ Antibodies |
| ☒ | ☐ Eukaryotic cell lines |
| ☒ | ☐ Palaeontology and archaeology |
| ☒ | ☐ Animals and other organisms |
| ☒ | ☐ Clinical data |
| ☒ | ☐ Dual use research of concern |
| ☒ | ☐ Plants |

## Methods

| n/a | Involved in the study |
| --- | --- |
| ☒ | ☐ ChIP-seq |
| ☒ | ☐ Flow cytometry |
| ☒ | ☐ MRI-based neuroimaging |

## Plants

| | |
| --- | --- |
| Seed stocks | *Report on the source of all seed stocks or other plant material used. If applicable, state the seed stock centre and catalogue number. If plant specimens were collected from the field, describe the collection location, date and sampling procedures.* |
| Novel plant genotypes | *Describe the methods by which all novel plant genotypes were produced. This includes those generated by transgenic approaches, gene editing, chemical/radiation-based mutagenesis and hybridization. For transgenic lines, describe the transformation method, the number of independent lines analyzed and the generation upon which experiments were performed. For gene-edited lines, describe the editor used, the endogenous sequence targeted for editing, the targeting guide RNA sequence (if applicable) and how the editor was applied.* |
| Authentication | *Describe any authentication procedures for each seed stock used or novel genotype generated. Describe any experiments used to assess the effect of a mutation and, where applicable, how potential secondary effects (e.g. second site T-DNA insertions, mosiacism, off-target gene editing) were examined.* |
