# Supplementary material for: A multicenter proof-of-concept study on deep learning-based intraoperative discrimination of primary central nervous system lymphoma
Source: Nat Commun. 2024 May 4;15:3768. doi: 10.1038/s41467-024-48171-x (PMC11069536; doi:10.1038/s41467-024-48171-x)
Supplement: Supplementary file 4 — Description of Additional Supplementary Files [file 41467_2024_48171_MOESM4_ESM.pdf]

Description of Additional Supplementary Files

Supplementary Movie 1:

A file containing video recorded that was used to show pathologists' decision-making process for differentiating PCNSL from glioma based on the deep learning model via the pathological decision support webpage.
